# COVID-19 Lockdown and Changes in Dietary and Lifestyle Behaviors in a French Longitudinal Cohort

**DOI:** 10.3390/nu15214682

**Published:** 2023-11-04

**Authors:** Antoine de Reviers, Agnès Helme-Guizon, Christophe Moinard, Catherine Féart

**Affiliations:** 1 University Grenoble Alpes, Inserm U1055, Laboratoire de Bioénergétique Fondamentale et Appliquée, SFR Structure Interdisciplinaire Grenobloise en Nutrition (SIGN), F-38000 Grenoble, France; antoine.dereviersdemauny@agroparistech.fr; 2 University Grenoble Alpes, Centre d’Etudes et de Recherche Appliqué à la Gestion (CERAG) & Grenoble Institutt d’Administration des Entreprises (IAE)-Institut National Polytechnique (INP), SFR SIGN, F-38000 Grenoble, France; agnes.helme-guizon@univ-grenoble-alpes.fr; 3 University of BordeauxBordeaux Population Health Research Center, Inserm, UMR 1219, F-33000 Bordeaux, France; catherine.feart-couret@u-bordeaux.fr

**Keywords:** lockdown, COVID-19, eating habits, sedentary behavior, physical activity, mental health, sleep, behavior change

## Abstract

Background: The COVID-19 pandemic has imposed local lockdowns resulting in strong disruptions in our lifestyles and dietary behaviors. This study aimed to determine how the lockdown in France affected these behaviors and weight during the lockdown and in a one month follow up period of time after the end of the lockdown. Methods: The study design was a longitudinal cohort, among French adults. A total of 593 participants (68.6% female), with a mean age of 42.2 years (SD = 15.2) completed a self-reported questionnaire on four occasions spaced one month apart, from the beginning of the lockdown starting 17 March 2020, until one month after its end (mid-June 2020). Clusters of participants were formed using the non-supervised k-means algorithm. Results: The mean weight gain after one month of lockdown was 0.56 kg (SD = 0.6). The cluster analysis exposed three different patterns of behavioral changes, despite no significant differences in age or BMI between clusters. These three groups have experienced different weight change dynamics over the follow-up duration. The first cluster (*n* = 210) reported fewer changes in sleep quality and quantity and less change in snacking frequency (*p* ≤ 0.001). The second cluster (*n* = 200) reported significantly lower levels of stress than the other clusters (*p* ≤ 0.001). The third cluster (*n* = 183) differed from the others, with a more degraded quality of sleep reported throughout the lockdown (*p* ≤ 0.01). However, changes in eating behaviors and body weight were not significant. Conclusions: During the lockdown, behavioral changes occurred, both health-favorable and non-health-favorable, yet they had a minor impact on eating behaviors and reported body weight once the restrictive measures were lifted. The identification of three patterns suggests that, in such constraining situations, personalized recommendations should be provided.

## 1. Introduction

The World Health Organization declared the coronavirus disease (COVID-19) pandemic as a global health emergency on the 30 January 2020 [1]. From then, COVID-19 confirmed cases began to sharply rise worldwide. To cope with, countries have established different approaches to reducing the rate of the virus propagation. The first peak of the health crisis occurred around March 2020, with varying timings depending on the countries. While different levels of restrictions were applied to populations, a lockdown was often used as a promising strategy to strongly enhance social distancing and thus limit the daily rate of COVID-19 infections. According to the United Nations Children’s Fund [2], 186 countries have applied several forms of movement restrictions related to COVID-19, including 82 countries which imposed either a full or partial lockdown. France was one of these countries, adapting its strategy to the severity of the health crisis. First, full lockdown restrictions were implemented on 17 March 2020; then, a partial easing of restrictions began on 11 May, with the full removal of restrictions on 2 June 2020. During the first period of full lockdown, French people were only allowed leaving their households for predetermined reasons, such as medical needs, essential work, practice of physical exercise, food purchases, and emergencies. People were also strongly encouraged to telework and/or study from home.

The conceptual framework motivating this study considers the lockdown a singular experimental setting common to the whole population. This trigger suddenly imposed major changes in lifestyles, such as limitations on free movement, restrictions on socializing, and drastic changes in access to food. This particular context was unprecedented, meaning that the impacts it could have on health factors such as psychological well-being, physical activity, and eating habits were still unexplored [3]. It was also impossible to predict whether the potential impacts would be acute or long-lasting, even after the restrictions had been lifted. Such abrupt and unexpected changes could have been detrimental for both mental health [4] and healthy lifestyle behaviors, in addition to the anxiety-related climate of the crisis. The potential underlying mechanisms are extensive and include factors such as psychological stress, disruptions in daily routines, social and environmental influences, and economic uncertainty. For instance, increased energy and alcohol consumption, markers of altered eating patterns, have already been reported [5]. Conversely, brutal modifications of daily routines have also promoted emotional disorders accompanied with appetite loss [6,7]. These controversial behaviors also resulted from various socio-environmental contexts as well as the internal resources (i.e., resilience) confined people had. To illustrate such changes, a systematic literature review performed on 35 cross-sectional studies and one cohort reported that in the post-lockdown period, compared with the period prior to lockdown, 11.1–72.4% of participants gained weight and 7.2–51.4% of participants lost weight (mainly observed among older adults >60 years old), associated with expected changes in BMI [8]. Focusing on dietary habits, the same group of authors also reviewed 32 studies and reported that the lockdown was associated with significantly increased snacking and alcohol consumption among up to 51.0% of the participants examined [9]. Regarding physical activity, several systematic literature reviews were conducted and collectively concluded that sedentary behavior increased among up to 16% of participants and physical activity decreased among up to 17% of participants during the full or partial periods of lockdown [10]. The published scientific literature has highlighted divergent outcomes in the assessment of the impact of lockdown measures on individuals’ behaviors, underscoring the complexity and variability of these effects [11]. Faced with the abundant literature on this topic, some studies included among these systematic reviews are documented in the present paper to better illustrate the available results in nine different countries (Supplementary Table S1).

Although few studies focusing on both diet and physical activity habits have been implemented, we can broadly conclude that lifestyle behaviors have been negatively modified worldwide; while some individuals adopted health-promoting behaviors, other subgroups adopted health-damaging behaviors during the lockdown [12].

Notably, most studies were only conducted once, providing snapshots of behavior before/after the lockdown, with both restrictions on mobility and sharing and several individual coping strategies being adopted during this first period of the health crisis. Indeed, few studies have been replicated after the lockdown, asking if people have re-turned to their pre-pandemic lifestyles partially or fully [13].

To address this gap, the primary objective of this study is to assess self-reported changes in several parameters of lifestyle, food intake, body weight, and psychological well-being over a four-month period starting with the first lockdown in France and ending up one month after restrictions were lifted. The secondary objective is to determine whether different patterns of change led to different body weight changes. Our initial hypothesis was that the period of lockdown introduced during the first wave of the pandemic in France would lead to significant changes in these parameters, which would last for up to a month after the restrictions were lifted. Furthermore, it was hypothesized that these variations in lifestyle parameters observed during this period were likely to vary according to the duration and specific nature of the restrictions applied, thus revealing the influence of these factors on changes in well-being indicators. Finally, it is possible that these variations in lifestyle parameters may be correlated with substantial changes in participants’ body weight, thus highlighting the existence of a potential link between fluctuations in lifestyles in response to confinement measures and bodily changes.

**Figure 1:**
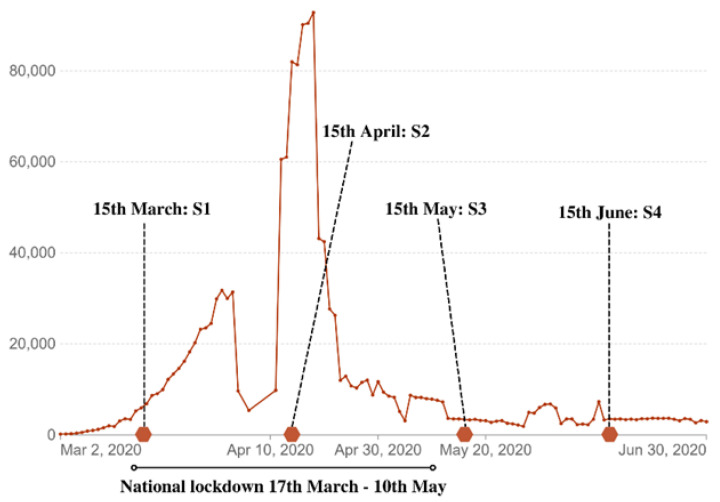
Weekly confirmed COVID-19 cases in France refer to the cumulative number of confirmed cases over the previous week [14]. The time points represent the collection periods of the different surveys: S1, S2, S3, and S4.

The research design of our study was longitudinal and involved a cohort of French adult participants. Data were collected at four different times, one month apart, beginning at the start of the lockdown (Figure 1). The analytical plan consisted firstly of a review of the data in the complete database, then analyses of the complete cohort (*n* = 593), and finally cluster analyses.

## 2. Materials and Methods

### 2.1. Study Design and Population Sample

The present study cohort was a longitudinal study based on an online survey of French adults. Data were repeatedly collected from the same sample over four successive rounds of collection using an updated questionnaire, launched roughly every month. Participants were informed at the time of the first questionnaire that there would be longitudinal follow-up, although they did not know when the next questionnaire would take place. Consequently, participants were invited to take part in each new questionnaire as the health context evolved. The first questionnaire was shared in mid-March, the second questionnaire was launched in the middle of the lockdown (mid-April), the third was launched one week after the partial removal of the re-strictions (mid-May), and the last one was launched one month after the end of the re-strictions (mid-June). The sampling methodology was a non-probability convenience methodology. The recruitment of participants was accomplished through a snowball effect, with the questionnaire being shared by the team’s professional and personal social networks, as well as by their professional email. Each person who received the link to the questionnaire had the opportunity to share it again.

### 2.2. Data Collection

Participants over 18 years of age were recruited through the researchers’ networks and through snowball techniques. Participants were invited to fill out the online questionnaire through a shared link. The study used the secure platform Sphinx. The initial set of questions included specific items to evaluate socio-economic parameters, perceived mental health, consumption of major food groups, snacking, changes in physical activity habits, and sedentariness. Both reported sleep quality and quantity were also collected. Finally, participants had to report their physical measurements and age (Figure 1).

The study was conducted according to the guidelines of the Declaration of Helsinki and complied with GDPR. All participants were explicitly informed at the beginning of each questionnaire and gave their consent for inclusion repeatedly. Participants were also informed that they had the right to withdraw from the study at any time during the study. All subjects were fully informed of the research’s objectives, and how their data would be processed and anonymized. All participants provided informed consent and an electronic email. The anonymity of each participant was guaranteed by a unique identification code that made them unidentifiable in the database. The email address was stored apart from the data collected.

**Figure 2:**
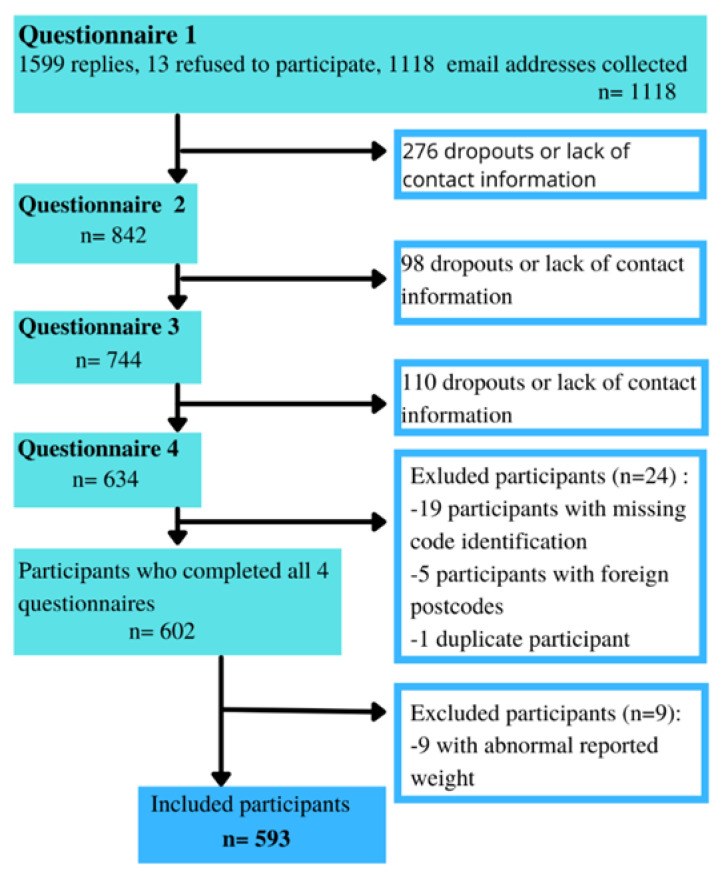
Flow diagram of the participant inclusion.

During the initial round of data collection (see Figure 2), we received a total of 1599 responses. However, only 1118 of these responses included a valid email address which allowed us to establish continued contact with the participants and incorporate them into the longitudinal follow-up. Subsequently, 276 participants were lost between the first and second questionnaires, 98 between the second and third, and 110 between the third and final questionnaires. The participant’s email address was used to ensure that the participant was a unique respondent; therefore, questionnaires collected without an email address had to be excluded. Finally, 41 participants were excluded at the time of the first database checks, 27 were missing anonymization codes, five had non-French postcodes, one participant was a duplicate, and nine participants reported body weight values that were not clinically plausible.

### 2.3. Questionnaires

The four shared questionnaires consisted of the same three sections: (1) perceived COVID-19 threat and stress; (2) current lifestyle (physical activity, sleep, and food hab-its); and (3) participants’ socio-demographic characteristics (marital status, number of children), household types (flat, house; with or without a garden/terrace), and current professional position (on-site work, remote work, or partial unemployment). Participants had to report their answers on four- or five-point scales, with a few exceptions. The questionnaire also included open-ended questions. Questions were adopted for each questionnaire in the surveyed period (at the beginning, during, and at the end of the lockdown). Participants were asked to report their answers for the answering period in comparison to the pre-lockdown situation (e.g., “*Compared to the pre-lockdown, TO-DAY, you eat less/as much/more of the following food items*”).

#### 2.3.1. Stress and COVID-19 Perceived Threat

Stress was evaluated using the validated Center for Epidemiologic Studies De-pression Scale (CES-D), which is a twenty-item scale [15]. The CES-D was designed for the general population and is now used as a screening tool for depression. It is based on twenty self-reported items scored on a four-point scale which measure the main dimensions of depression experienced over the past week. This scale has been tested on multiple populations of different genders and cultures and maintains consistent validity, reliability, and internal consistency (Cronbach’s alpha ≥ 0.9) [16,17,18]. The survey also measured the perceived severity of and personal vulnerability to the COVID-19 threat following the method of Rosenstock et al. (1988), with two items on five-point scales assessing, respectively, the perceived severity and perceived vulnerability [19].

#### 2.3.2. Physical Activity

Both physical activity and sedentary behaviors were assessed. In the first questionnaire (S1), participants were asked to report their current daily physical activity time compared to before the lockdown. The three following questionnaires (S2, S3, and S4) evaluated the type of physical activity as well as its modalities (alone, in a group, or online).

#### 2.3.3. Sleep

In view of the inherent variability in individual sleep patterns, we chose to measure differential sleep variations from individual baselines in our study. Self-reported sleep quality and quantity were also measured on a scale comparing the perceived rating at the time of the questionnaire in comparison to the pre-lockdown period. This approach provides a stable reference point, considering individual variability, and makes it easier to assess the specific impact of the lockdown on each participant’s sleep.

#### 2.3.4. Dietary Habits

Food consumption was evaluated for a range of food categories (vegetables and fruits, local and in-season products, sweet drinks, alcohol, fatty foods, processed/home-made food, organic food, pasta/rice, and others) on a five-point scale ranging from “much lower consumption” to “much higher consumption” in addition to a “no consumption” modality. Additionally, participants were asked to report their variation in snacking frequency.

#### 2.3.5. Social Demographics and Other Personal Data

The last section of the survey included questions on each participant’s socioprofessional category, employment status, demographics, sex, age, height, and body weight.

### 2.4. Statistical Analyses

The principal objective of this research was to identify patterns in lifestyle changes over a four-month period. Thus, firstly, we factorized food eating behavior categories. Based on the food-related items within the questionnaire, factor scores for healthy eating were created using principal component analysis (PCA). The same approach was used to calculate an unhealthy diet score based on the related items. We reduced these variables into two dimensions, “healthy pattern” and “unhealthy pattern”, which alone retained more than 83% of the variance. The “healthy pattern” factor score included the items “fruits”, “home-made”, “organic”, and “local products”. The “unhealthy pattern” factor score included the items “soda”, “sugars”, “snacks”, “alcohol”, “processed foods”, and “fatty products”. All diet-related variables were re-categorized before analysis as follows: 1 (decreased intake compared to last month), 2 (unchanged intake compared to last month), and 3 (increased intake compared to last month). Factor scores were composed based on principal component analysis. All statistical analyses were performed using SPSS Statistics 21.0 (IBM SPSS Statistics for Windows, Version 28.0. Armonk, NY, USA: IBM Corp.) and RStudio (RStudio Team (2020). RStudio: Integrated Development for R. RStudio, PBC, Boston, MA, USA) [20,21].

To study participants’ behaviors throughout the four months of follow-up, clusters of similar behaviors were formed from the responses to the first questionnaire. Each participant was assigned to a cluster based on the behaviors collected in the first questionnaire. Clusters were determined using eleven parameters: level of mental stress, variation in mental stress, physical activity, sedentariness, sleep quantity, sleep quality, socio-professional category, age, and BMI. These clusters were made using an unsupervised iterative machine-learning algorithm (K-means clustering). To avoid influencing the initialization of the algorithm by selecting an a priori number of clusters, the optimal number of K-clusters was determined according to scree plots (elbow method) and silhouette scores. The results showed that k = 3 was the optimum for minimizing the within-group sum of squares while maximizing the between-group variance. The pro-files obtained revealed two main dimensions explaining, respectively, 18.1% and 15.9% of the total variance.

Statistical tests were then performed to determine if there were significant differences between the groups during the four follow-up periods (S1, S2, S3, and S4). Because most of the variables did not follow a normal distribution, nonparametric tests (Kruskal–Wallis) were used, and post hoc tests were performed to adjust for multiple comparisons (Bonferroni test). Moreover, the chi-squared test was used to assess the differences between clusters.

## 3. Results

### 3.1. Participant’s Characteristics

After initial database cleaning, the study population consisted of 593 participants (68.6% female) with a mean age of 42.2 years (SD = 15.2) and a mean BMI of 23.4 (SD = 4.32) at inclusion (S1). Further descriptive characteristics of the population are detailed in Table 1. We did not observe any significant differences in these three parameters (gender, age, and BMI) for respondents to the first questionnaire (*n* = 1599) compared with participants who completed all four questionnaires. We therefore assume that the gradual attrition observed across the four waves of participant responses can primarily be attributed to a subset of participants categorized as “lost to follow-up” and that it does not introduce a selection bias into our sampling process.

### 3.2. Whole Cohort’s Lifestyle Evolution

**Figure 3:**
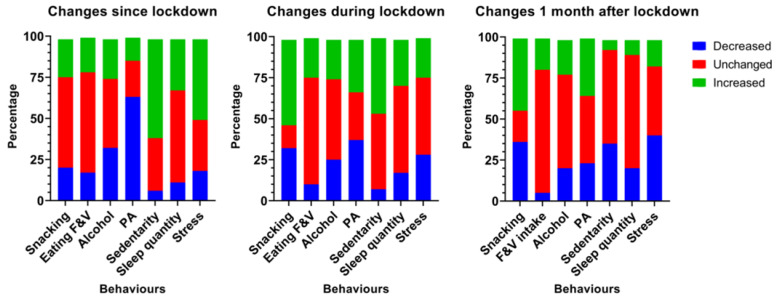
Evolution of the main changes in behaviors at the start of the lockdown, one month after (during) the lockdown, and one month after the measures were lifted. F&V: fruits and vegetables, PA: physical activity.

Initially, an analysis of behavioral and lifestyle variations was conducted for the entire cohort, comprising all participants who successfully completed all four questionnaires (*n* = 593). On average, the mean weight gain after one month of lockdown was 0.56 kg (SD = 0.6). However, this mean does not reflect the behavioral disruptions that partly explain the mechanisms underlying the weight variations. These lifestyle pat-terns are shown in Figure 3.

Initially, it was found that the onset of the lockdown led to an acute decrease in physical activity levels and an increase in sedentary behaviors in more than two-thirds of the cohort. The reported level of mental stress was higher in half of the population. The frequency of snacking and the consumption of fruit and vegetables remained stable in more than half of the cohort. However, after one month of lockdown, more than 55% of the sample reported that they had increased their snacking frequency. One month after the end of the restrictions, one third of the participants reported a decrease in their level of sedentary behavior, but a majority reported no change.

**Figure 4:**
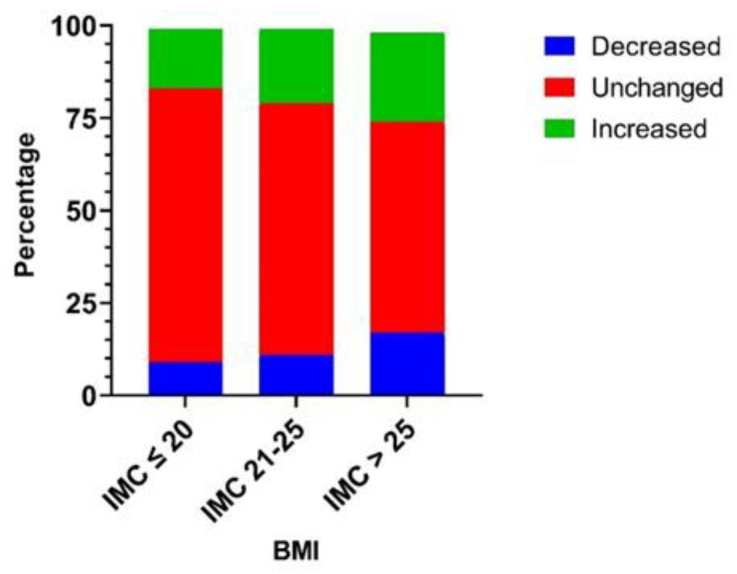
Reported modification of food intake amount classified by participants’ Body Mass Index (Body weight(kg)/height(m)^2^). The association between food intake amount alteration and BMI class was tested using a chi-squared test and was statistically significant (*p* = 0.0459). Participants with a higher BMI (>25 kg/m^2^) were more likely to report a modified food intake amount than participants with low- and normal-range BMI scores.

Among this cohort (*n* = 593), we previously observed that individuals with lean (<18.5) or overweight (25–30) BMIs had higher odds of experiencing BMI changes (±0.5) by the end of the lockdown. Subsequently, the results presented in Figure 4 indicate that overweight individuals (BMI greater than 25) had more statistically significant changes in their food intake quantity, either downward or upward, compared to people with lower BMI scores [22].

### 3.3. Clusters Compositions

The clusters were formed based on the entire cohort included in the analysis (*n* = 593), that is, all the participants who completed the four questionnaires. The obtained clusters were distributed as follows: 210 participants in cluster 1 (C1), 200 participants in cluster 2 (C2), and 183 participants in cluster 3 (C3). No significant difference in age or BMI was found between the clusters. Cluster 3 had a slightly higher proportion of women than the other two clusters, i.e., 77.6% in comparison to 66.2% (C1) and 63.0% (C2). The Mahalanobis’ distance results (Table 2) indicate the absence of multivariate outliers in the model, as each distance is below the critical distance of eight degrees of freedom and the significance level set at 0.05 (15.507).

### 3.4. Behavioral and Lifestyle Change Paths over the Course of the Lockdown and Afterward

**Figure 5:**
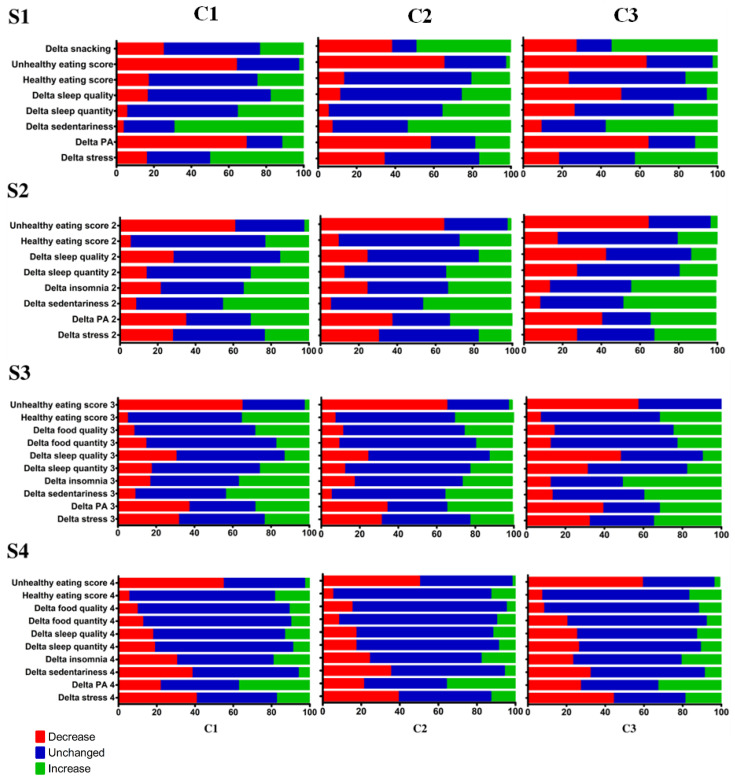
Cross-table of behavioral variations over time among the three clusters. “C” represents the clusters and “S” represents the survey. Data are expressed as the percentage of participants who reported a decrease, increase, or stable behavior for each item.

Among the three clusters composed, different pathways emerged (Figure 5). The imposed disruptions provoked diverse responses from individuals, varying across the four studied periods.

In Period S1, at the initiation of the lockdown, participants in cluster 1 maintained relatively stable snacking habits, in contrast to a majority who reported snacking more and a high proportion who reported snacking less. The created scores for “healthy” and “unhealthy” diets did not show significant differences. Regarding the sleep items, cluster 3 was significantly different from the other two clusters because it more frequently reported a decrease in both sleep quality and quantity. Furthermore, over this period, variations in stress differed between the three clusters (*p* < 0.001). Significance levels are shown in Table 3.

As for the S3 period, cluster C3 stands out for significantly larger sleep quantity and quality disturbances compared to clusters C1 and C2. Cluster C2 was also more likely to report an increased level of stress compared to the first S1 questionnaire. Variations in physical activity were similar within the three clusters: about 40% of the subjects re-ported a decrease in physical activity, while another 30% reported more physical activity than at the beginning of the lockdown. In addition, the “healthy eating” and “unhealthy eating” factor scores differed little between participants, with a tendency for cluster C3 to report a decrease in consumption categorized as “healthy eating”. Concerning the S3 period, the three clusters showed a decrease in consumption of “unhealthy” products, in concordance with the previous questionnaires, S1 and S2. All three clusters reported similar behavioral changes, except for the third cluster which reported a decrease in both sleep quality and quantity twice as frequently as the other clusters. In addition, a high proportion of participants reported an increase in the consumption of healthy products among the three clusters.

Finally, the results for the S4 period indicated similar changes between the three clusters. The only parameter that was significantly different was the change in the number of reported insomnia cases, which was slightly greater in cluster 3. In general, the three groups reported mostly unchanged behaviors from the previous month. Only the “unhealthy eating” item showed strong variations, with about half of the participants declaring to have decreased their consumption of these products. Stress levels or sedentary behaviors also decreased or stabilized for over 80% of the three clusters.

All three clusters showed the same trend of highly disturbed behaviors at S1, which gradually faded month after month, leading to more stability in the last questionnaire after three months of follow-up.

**Figure 6:**
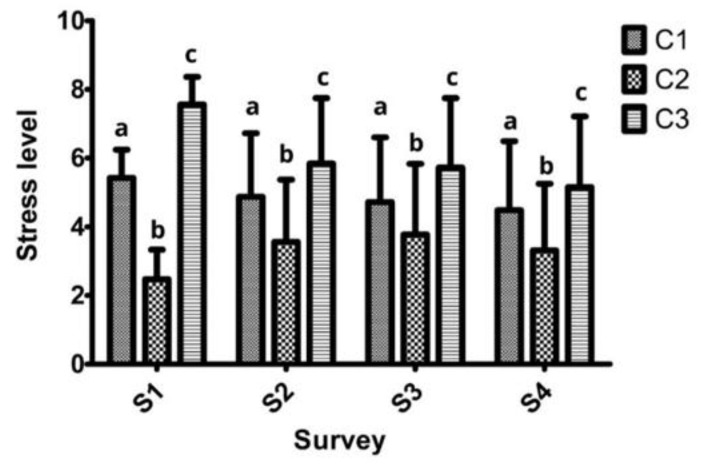
Reported stress levels on a ten-level scale at S1, S2, S3, and S4. Values are means (standard deviation). Significantly different group means are represented by different letters (a, b, and c) and were compared independently for each survey. Test performed: one-way ANOVA. Normality was assessed using the D’Agostino–Pearson test and multiple comparisons were adjusted using a Tukey test.

The results of the stress levels (Figure 6) on a scale of one to ten show significant differences between the three clusters. Participants in cluster C3 reported the highest levels of stress during the four-month period. Interestingly, in clusters C1 and C3, the reported levels of stress decreased continuously during this longitudinal study. Participants in cluster C2 exhibited the lowest levels of stress. However, they were higher in the one-month period after the end the lockdown than they were at the onset of the lockdown, in contrast to cluster C1 and cluster C3, which followed an opposite trend.

**Figure 7:**
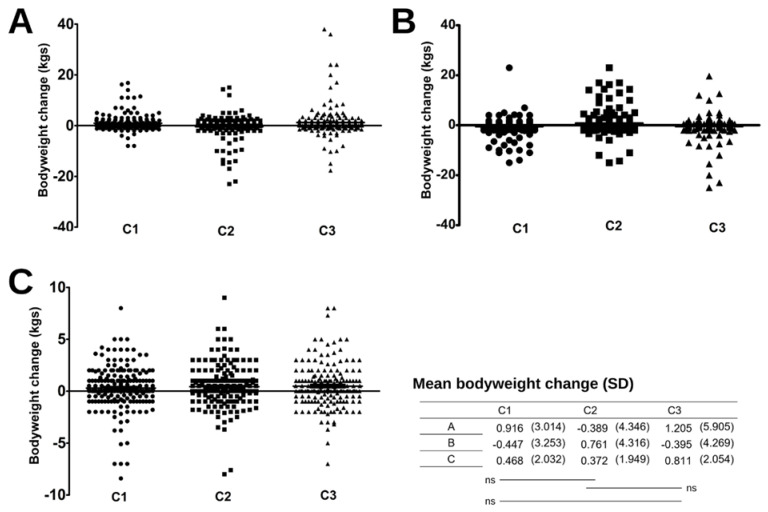
Scatter plot of the change in reported body weight between the beginning and end of lockdown (**A**), between the lockdown and one month after the removal of the restrictions (**B**), and during the entire follow-up period (**C**). Each point, square, and triangle represent a participant from clusters C1, C2, and C3 respectively. Statistical differences were tested by a Kruskal-Wallis test (ns: not significant).

The mean body weight change (Figure 7) was not significantly different between groups, whether during the lockdown period, after the removal of restrictions, or during total monitoring (between S1 and S4). However, the three clusters seem to have followed different weight evolution dynamics during the three months of follow-up. Thus, while clusters C1 and C3 reported an average gain of about 1 kg, cluster C2 lost an average of 0.39 kg (SD = 4.34) at the onset of the lockdown. Subsequently, C1 and C3 have lost weight on average during lockdown, whereas C2 gained 0.76 kg (SD = 4.31) on average. However, at the end of the follow-up, these differences stabilized and the participants of the three groups did not show statistically significant differences in weight on average.

## 4. Discussion

The primary objective of this longitudinal study was to describe the evolution of both the nutritional status, the dietary habits, the practice physical exercise and the mental health of confined French people, up to three times during the period of lockdown and once after. The secondary objective was to determine whether different patterns of change led to different body weight changes. Aggregate analyses of the full cohort (*n* = 593) indicate a clinically insignificant mean weight gain either at the end of lockdown or one month after its completion. Our results suggest that variations in lifestyle behaviors, however important, have had a limited impact on body weight changes. Our results are in line with another study evaluating changes in eating habits in France and possible changes in food behavior and lifestyle in France among the general population [23] or students [24] and close countries [25].

More specifically, the cluster analysis revealed three different patterns of behavioral changes. Cluster C1 experienced an average weight gain of close to one kilogram over the lockdown period. Participants in this cluster also reported fewer changes in sleep quality and quantity and fewer changes in snacking frequency. This cluster’s average weight gain was partially offset by an average weight loss of about half a kilogram when restrictions were lifted. Interestingly, this group reported less variation in snacking frequency but did not have significantly different results for the “healthy eating” and “unhealthy eating” composite scores. This may suggest that snacking variations did not significantly affect dietary intake.

Cluster C2 reported significantly lower levels of mental stress than the other clusters and was also more likely to report a decrease in stress levels at the onset of the restrictions. This group could be characterized as more resilient. On the question of mental well-being and anxiety, the WHO reported an increase in the prevalence of anxiety disorders of 25% worldwide in 2020 as a result of the pandemic [26]. Studies have also suggested that certain population sub-groups are more likely to develop this increase in anxiety associated with the pandemic, particularly women and young adults [27]. In terms of weight changes, this cluster experienced a slight decrease in average body weight (−0.39 kg) during lockdown. This result, which may seem counterintuitive, is consistent with the literature, which has often reported groups of individuals exhibiting behaviors opposite to the general trend and decreases in body weight [28,29]. However, this group experienced the greatest average weight gain (0.76 kg) following the lockdown. This could potentially be explained by a rapid return to pre-lockdown habits.

The third cluster differed from the others due to a more degraded quality and quantity of sleep reported throughout the lockdown and an even greater increase in the level of insomnia one month after the end of the imposed measures. This group also reported higher levels of stress than the other two groups throughout the follow-up. In terms of body weight, cluster 3 showed an average increase of 1.2 kg during lockdown, which stabilized at 0.8 kg one month after the end of restrictions. These disturbed sleep outcomes that are sustained over time are clinically important and may represent an increased risk, as the context of sleep restriction has been associated with weight gain, abdominal fat, and increased caloric intake [30]

Overall, since our variables measured behaviors in dynamic ways (i.e., increasing, decreasing, or stable), one could have observed that strong disruptions in lifestyles at the beginning of lockdown was followed by opposite changes at the end of lockdown back to their initial state. Overall, since our variables measured behaviors in dynamic ways (i.e., increasing, decreasing, or stable), one could have observed that strong disruptions in lifestyles at the beginning of lockdown were followed by opposite changes at the end of lockdown, when lifestyles returned to their initial state. However, this pattern was hardly discernible, which suggests that the different behaviors examined returned to their initial levels progressively over time and not directly upon lifting the restrictions. Alternatively, some behavioral changes were maintained over time by creating new “healthy” or “adverse” habits. Thus, our results suggest that despite the strong disruptions in lifestyles, body weight underwent only moderate variations, suggesting that it is the result of a complex system. The few published studies with a design similar to this work have emphasized that different patterns of behavior change reflect socioeconomic inequalities [12]. Moreover, the most health-promoting behavior changes were generally obtained in cohorts composed of a healthy population with high socioeconomic status [31] or older adults [32].

The disrupted food availability, disrupted social relations, and uncertainty created by the lockdown can be viewed as stressors that align with our conceptual framework. These stressors may have led to emotional and psychological distress [33]. In this regard, it is possible that people who benefited from privileged isolation conditions, with little impact on access to food, preserved social contacts, and a relatively low sense of vulnerability, were less affected psychologically, resulting in eating, sleeping, and physical activity behaviors that were relatively stable compared to their pre-isolation levels.

### Strength and Limitations

A weakness of this study is the use of self-reported data, a mode of data collection forced by the health context at the time (but the same limitation applies to the majority of studies similar to ours). Self-reported data are subject to social desirability bias, and participants may have under-reported certain socially under-valued behaviors or over-reported socially valued behaviors. However, the qualitative material we collected (open-ended questions) suggests that most of the participants were committed to the study and motivated to provide accurate answers.

Moreover, the results must be interpreted in the light of the socio-demographic context of the study population. Our sample is not intended to be representative of the entire French population; rather, it describes the changes observed in a particular framework, in a population mainly from the Grenoble area, France, with a high level of education and a relatively low rate of overweight compared to the French population [34].

The nature of this study is observational, and this study’s intrinsic design limitations, which preclude controlled experiments or interventions, compel us to approach causality with caution. Rather than affirming direct cause-and-effect relationships, our study’s value lies in its capacity to illuminate associations between variables, thereby affording a foundation for the formulation of hypotheses and guiding future research endeavors. In recognition of the inherent constraints of observational studies, it is imperative to conscientiously consider these limitations when interpreting the ramifications of our findings within the broader academic literature.

This work is also one of the few to have followed participants longitudinally over time, which could reduce memory bias compared to studies that interview subjects over two distinct periods within a single questionnaire.

## 5. Conclusions

In this study, we have provided data on a French population sample’s lifestyle, eating habits, level of stress, and sleep patterns. Our study offered a longitudinal design with four data collection time points, from the first phase of lockdown to one month after the end of lockdown.

Since the COVID-19 crisis has continued to impose repeated intermittent local lockdowns to date, it is important to identify which populations are most impacted and which lifestyles and behaviors are the most likely to change during these episodes. Enhanced support should be directed at these populations to prevent adverse health effects. These findings could be of particular importance for future public health policies and strategies in emergency scenarios such as a pandemic or any crisis or major disruption imposed on our way of life.

## Figures and Tables

**Table 1:** Characteristics of the study population.

Variables		Total 100%	Total *n* = 593
Gender	Female	68.6	407
	Male	31.4	186
Age (years)	18–29	24.8	147
	30–49	43.2	256
	50–69	27.0	160
	>70	5.2	31
BMI	Underweight (<18)	5.4	32
	Normal (18–25)	70.0	415
	Overweight (25–30)	18.2	108
	Obesity (>30)	6.4	38
Professional activity during the lockdown	Home working	46.5	276
	Working on-site	10.1	60
	Technical or partial unemployment	12.1	72
	Others	17.7	105
Socio-professional category	Farmers operators	0.0	0
	Craftsmen, traders, and entrepreneurs	1.7	10
	Managers and higher intellectual professions	31.2	185
	Intermediate professions	3.2	19
	Employees	12.3	73
	Manual workers	0.3	2
	Retired	5.4	32
	Students	12.5	74
	Other persons without professional activity	33.4	198
Family composition	Alone	9.9	59
	Couple	38.1	226
	Housemates	0.7	4
	Others	40.5	240
	Children	10.8	64
Children	None	72.7	431
	1–2	22.1	131
	≥3	5.2	31
Type of dwelling	With garden	63.2	375
	Without garden	33.6	199
	Others	3.2	19
Daily PA before lockdown (minutes)	<15	10.5	62
	15–29	25.0	148
	30–44	23.4	139
	45–60	18.9	112
	60–90	12.6	75
	>90	9.6	57

**Table 2:** Mahalanobis distances between the final cluster centers. Performed with eight degrees of freedom and eight predictive variables in the model.

Cluster	1	2	3
1		3.049	2.403
2	3.049		5.334
3	2.403	5.334	

**Table 3:** Significance levels for differences between clusters across the four surveys. Differences were tested using a Kruskal–Wallis test and adjusted using a Bonferroni test for multiple comparisons. The colors indicate the trend of the *p*-value.

	Item	*p*-Value					Item	Sig.		
		C1-C2	C1-C3	C2-C3				C1-C2	C1-C3	C2-C3
S1	Delta snacking	0.000	0.000	0.195		S3	Unhealthy eating score 3	1.000	1.000	0.638
	Unhealthy eating score	1.000	1.000	0.894			Healthy eating score 3	1.000	1.000	1.000
	Healthy eating score	1.000	0.094	0.122			Delta food quality 3	1.000	0.695	1.000
	Delta sleep quality	0.474	0.000	0.000			Delta food quantity 3	0.840	0.496	1.000
	Delta sleep quantity	0.949	0.000	0.000			Delta sleep quality 3	1.000	0.001	0.000
	Delta sedentariness	0.001	0.011	1.000			Delta sleep quantity 3	1.000	0.001	0.002
	Delta PA	0.013	0.862	0.259			Delta insomnia 3	1.000	0.810	1.000
	Delta stress	0.000	0.000	0.000			Delta sedentariness 3	1.000	1.000	1.000
							Delta PA 3	1.000	0.726	1.000
S2	Unhealthy eating score 2	0.832	1.000	0.586			Delta stress 3	1.000	0.170	0.136
	Healthy eating score 2	1.000	0.084	0.035						
	Delta sleep quality 2	1.000	0.035	0.007		S4	Unhealthy eating score 4	1.000	1.000	1.000
	Delta sleep quantity 2	1.000	0.000	0.000			Healthy eating score 4	1.000	1.000	1.000
	Delta insomnia 2	1.000	0.689	0.390			Delta food quality 4	1.000	1.000	1.000
	Delta sedentariness 2	1.000	1.000	1.000			Delta food quantity 4	0.337	0.265	1.000
	Delta PA 2	1.000	1.000	1.000			Delta sleep quality 4	1.000	0.779	0.273
	Delta stress 2	0.720	0.398	0.027			Delta sleep quantity 4	1.000	1.000	0.378
							Delta insomnia 4	1.000	0.032	0.002
							Delta sedentariness 4	1.000	0.058	0.141
							Delta PA 4	1.000	0.891	0.463
							Delta stress 4	1.000	1.000	1.000

## Data Availability

The data presented in this study are available on request from the corresponding author.

## Supplementary Materials

The following supporting information can be downloaded at: https://www.mdpi.com/article/10.3390/nu15214682/s1, Table S1: Non-exhaustive summary of the main dietary and lifestyle behaviors changes related to the lockdown observed worldwide, specific to a single country. References [12,28,35,36,37,38,39,40] are cited in the Supplementary Materials.
