# Supplementary material for: COVID-19 Lockdown and Changes in Dietary and Lifestyle Behaviors in a French Longitudinal Cohort
Source: Nutrients. 2023 Nov 4;15(21):4682. doi: 10.3390/nu15214682 (PMC10648805; doi:10.3390/nu15214682)
Supplement: Supplementary file 1 [file nutrients-15-04682-s001.zip › nutrients-2641169-supplementary.pdf]

Authors	Design	Sample	Age	Zone	Gender	Period	Changes in PA (%)			Changes in BW (%)			Changes in food consumption (%)			Mental health
		n	years		%		Decreased	Unchanged	Increased	Decreased	Unchanged	Increased	Decreased	Unchanged	Increased	
Cheikh Ismail et al.	Cross-sectional	1012	18-35 : 56.8%<br>>35 : 53.2%	UAE	M: 24<br>F: 76	Between April and May 2020	41.9	43.3	14.8	21	48	31	The percentage of participants consuming breakfast increased from 66% to 74.2%. and the percentage of those skipping meals decreased from 64.5% to 46.2% during the pandemic			During vs pre 14.1% vs. 6.3% for all the time emotional exhaustion; 13.5% vs. 6.9% for irritability
Deschasaux et al.	Longitudinal	37752	52 (16.6)	France	M: 48<br>F: 52	Early april - May 2020	52.8	28.5	18.7	23	42	35	33	42	25	10.8% reportred modified eating practices (eating more) due to more anxiety
Giacalone et al.	Cross-sectional	2642	18-35: 35.3%<br>36-50: 37.2%<br>51-65: 23.5%<br>65+ 4%	Danish adults	M: 29<br>F: 71	From 24 April to 5 May 2020	48.8	21.5	29.7	49.4		28.4	57.2		42.8	–
Huber et al.	Cross-sectional	1964	23 ± 4	Bavarian unis.	M: 28.5<br>F: 71.5	Active for 2 weeks in March and April 2020	44.5	22.7	32.8	–	–	–	17	52	31	35.4 participants with increased mental stress
Reyes-Olavarra et al.	Cross-sectional	700	31 (18-62)	Chili	M: 25<br>F: 75	May and June 2020	M: 51.2<br>F: 58.7	M: 21.5<br>F: 21.4	M: 27.3<br>F: 19.9	M: 19.8<br>F: 14.2	M: 54.6<br>F: 47.7	M: 25.6<br>F: 38.1	–	–	–	–
Robinson et al.	Cross-sectionnal	2002	35 (12.3)	UK	M: 38<br>F: 62	28th April-22nd May 2020	40	15	45	–	–	–	22.6 less snacking	21.7 same snacking	55.7 more snacking	58% felt more anxious
Scarmozzino and Visoli	Cross-sectional	1932	63% between 21 and 35<br>21% 36-65<br>14% less than 20 years old	Italy	M: 33<br>F: 67	3 April 2020 to 15 April 2020	–	–	–	29.8	50.7	19.5	-	47.1	52.9	42.7% percent of this cohort attributed comfort food increase to higher anxiety levels
Gonzalez-Calderon et al.	Cross-sectional	2834	41 (10.50)	Spain	M: 31<br>F: 69	19 May to 31 May 2020	M: 63.6<br>F: 57.0	M: 16.0<br>F: 11.8	M: 20.5<br>F: 31.3	M: 28.6<br>F: 21.2	M: 36<br>F: 36.6	M: 35.4<br>F: 42.2	M: 20.1<br>F: 15.5	M: 41.4<br>F: 38.3	M: 38.5<br>F46.1	Anxiety M: 52.9<br>F: 68.1

Supplementary Table S1. Non-exhaustive summary of the main dietary and lifestyle behaviors changes related to the lockdown observed worldwide, specific to a single country. *UAE: United Arab Emirates, unis: universities.*
